# Supplementary figures and images for: Increased NFAT and NFκB signalling contribute to the hyperinflammatory phenotype in response to Aspergillus fumigatus in a mouse model of cystic fibrosis
Source: PLoS Pathog. 2025 Feb 4;21(2):e1012784. doi: 10.1371/journal.ppat.1012784 (PMC11957335; doi:10.1371/journal.ppat.1012784)

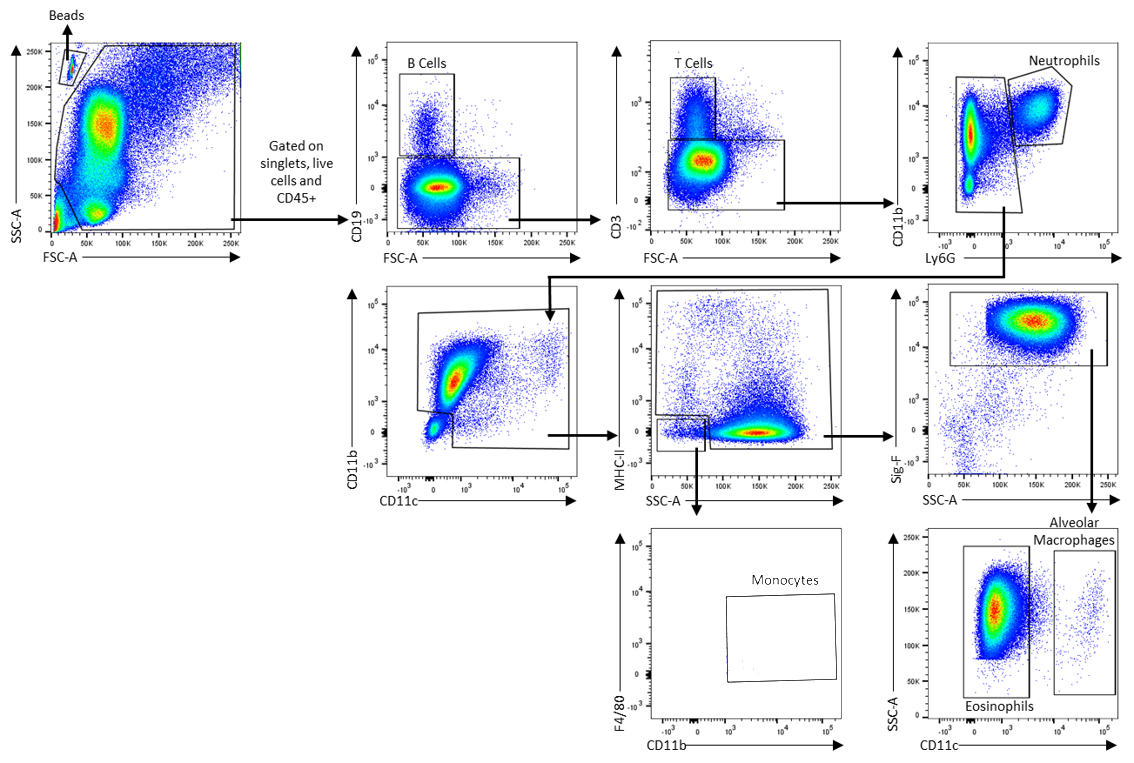

Supplement: S1 Fig — Cells were first gated on forward and side scatter followed by the exclusion of doublets. Dead cells were removed from analysis according to a LIVE/Dead stain and immune cells were identified as CD45+, cell types were subsequently determined through positive and negative getting of cell specific markers. Cell populations were enumerated using flow cytometry counting beads, identified by forward and side scattered, followed by autofluorescence. (TIF) [file ppat.1012784.s001.tif]
