# Supplementary material for: Increased NFAT and NFκB signalling contribute to the hyperinflammatory phenotype in response to Aspergillus fumigatus in a mouse model of cystic fibrosis
Source: PLoS Pathog. 2025 Feb 4;21(2):e1012784. doi: 10.1371/journal.ppat.1012784 (PMC11957335; doi:10.1371/journal.ppat.1012784)
Supplement: S1 Table — (DOCX) [file ppat.1012784.s002.docx]

|  | Healthy Control (*n*=6) | Cystic Fibrosis (*n*=6) |
| --- | --- | --- |
| Gender |  |  |
| Male n (%) | 3 (50.0) | 3 (50.0) |
| Female n (%) | 3(50.0) | 3 (50.0) |
| Age (Years), Average ± SD | 32.8 ± 7.9 | 27.3 ± 14.6 |
| CFTR mutation, *n* (%) |  |  |
| delta F508 | N/A | 6 (100) |

**Supplementary Table 1: Human donor demographics**
